# A79 PATIENT AND PHYSICIAN PERSPECTIVES ON IMPLEMENTION OF BEDSIDE INTESTINAL ULTRASOUND FOR THE ASSESSMENT OF INFLAMMATORY BOWEL DISEASE

**DOI:** 10.1093/jcag/gwad061.079

**Published:** 2024-02-14

**Authors:** R Chan, M Stewart, J Jones

**Affiliations:** Dalhousie University Faculty of Medicine, Halifax, NS, Canada; Dalhousie University Faculty of Medicine, Halifax, NS, Canada; Dalhousie University Faculty of Medicine, Halifax, NS, Canada

## Abstract

**Background:**

New tools for evaluating Inflammatory Bowel Disease (IBD) activity and complications are needed to deliver timely disease management and improve patient outcomes. Point of care (POC) intestinal ultrasound (IUS) is safe, inexpensive, and less invasive compared to standard endoscopy and imaging.

**Aims:**

To evaluate the implementation of POC IUS within an ambulatory IBD clinic at the QEII Health Sciences Center.

**Methods:**

This was a retrospective evaluation of a cohort of patients who had undergone IUS within the Nova Scotia Collaborative Inflammatory Bowel Diseases Clinic (NSCIBD) between January 18, 2023 and August 13, 2023. Medical records were reviewed retrospectively. Patient demographics, disease-related characteristics, perceived impact of IUS on need for endoscopy, perceived impact of IUS on IBD management, and clinical utility of IUS were collected. Surveys were administered prospectively to referring care providers and patients following IUS. Patient surveys assessed IUS satisfaction, acceptance of other methods to assess IBD activity, whether IUS improved knowledge of illness, and impact on quality of life (QoL).

**Results:**

One physician completed 107 IUS exams on 92 patients at the NSCIBD clinic over a nine-month period. Of 92 patients, 47 (51%) were male and 78 (85%) had Crohn’s disease. 53 patients provided an email and consented to survey participation. 34 responses were submitted (64%). There were 97 responses (91%) from 12 referring gastroenterology specialist clinicians.

Ninety-three percent of responses show IUS reports were very helpful (60%) or somewhat helpful (33%). More than a third (37%) of IUS exams were reported to delay or prevent the need for endoscopic evaluation, and 34% were reported to prompt a therapeutic change.

Of available tools, IUS had the highest proportion of patients who felt it was completely acceptable (77%), compared to colonoscopy (41%), stool sampling (50%), blood tests (71%), and computed tomography/magnetic resonance imaging (47%). Most (89%) indicated IUS caused either no discomfort (68%) or little discomfort (21%), with no patient reporting a lot of discomfort. Some patients (41%) reported that IUS improved their understanding of their illness, and 38% believe it improved their QoL.

**Conclusions:**

POC IUS offers an accurate, less invasive option for monitoring IBD activity compared to traditional investigations. There is limited data on implementation of, and patient and clinician perspectives regarding use of IUS and its clinical implications. Even early in implementation, POC IUS has clear benefits. This study suggests that IUS is highly acceptable to patients for investigating/monitoring their illness. Treating clinicians find significant value in IUS and report that it can prevent the need for endoscopic evaluation and prompt changes in therapeutic management.

**Funding Agencies:**

None

